# Opening the Window to Cancer: Potential Mechanism behind Increased Susceptibility in Rats Exposed Prenatally to BPA

**DOI:** 10.1289/ehp.118-a490b

**Published:** 2010-11

**Authors:** Julia R. Barrett

**Affiliations:** **Julia R. Barrett**, MS, ELS, a Madison, WI–based science writer and editor, has written for *EHP* since 1996. She is a member of the National Association of Science Writers and the Board of Editors in the Life Sciences

Exposure to environmental factors before birth or during other critical periods of development can cause subtle changes in a tissue’s molecular foundations, leading to health effects later in life. Prenatal exposure to bisphenol A (BPA) is linked to cellular and structural changes in the mammary glands of adult rats and increased susceptibility to chemically induced cancer. New research now suggests a possible mechanism of action for this increase in cancer susceptibility via altered protein expression patterns in rat mammary gland tissue **[***EHP* **118(11):1614–1619; Betancourt et al.]**.

Pregnant rats were exposed to 0, 25, or 250 μg BPA/kg/d from day 10 to 21 postconception, and their female offspring were given a single dose of the cancer-inducing compound dimethylbenzanthracene (DMBA) at 50 or 100 days after birth (i.e., young adulthood). Experiments were conducted to determine the relationship between prenatal BPA exposure and the expression of proteins intrinsic to the growth and development of the mammary gland in adulthood. These proteins included estrogen receptor-α (ER-α); PR and Bcl-2, downstream targets of ER-α; steroid receptor coactivators (SRCs) 1 to 3, which influence ER-α transcriptional activity; and several growth factor receptors and signaling molecules that direct cell proliferation and programmed death.

Body weight and hormonally sensitive end points (time to vaginal opening, serum levels of the hormones 17β estradiol and progesterone, and estrous cyclicity) were assessed but found to be unchanged in relation to prenatal BPA exposure. ER-α, PR, and Bcl-2 were significantly downregulated and SRC-3 and some signaling molecules were upregulated in rats exposed prenatally to BPA.

DMBA administered to rats at 50 days did not yield significant differences in tumor incidence between those with or without prenatal BPA exposure. Among animals that received DMBA at 100 days, all assayed proteins were significantly upregulated in the BPA-exposed rats, and cell proliferation was enhanced, but apoptosis was unchanged. These animals also had decreased time to tumor formation and increased tumor incidence and severity.

These findings suggest that prenatal exposure to BPA in rats alters expression of key receptors and components of cellular signaling pathways in the mammary gland in adulthood, consequently increasing the tissue’s susceptibility to chemically induced cancer. Whether this occurs in humans is unknown. Data from the Centers for Disease Control and Prevention indicate human exposure to the chemical is widespread, with approximately 95% of Americans estimated to have detectable levels of BPA metabolites in their urine. Continued research is necessary to elucidate the mechanisms by which BPA exposure early in life influences the development of permanent effects in maturity.

